# Thiouracil in Thyrotoxicosis

**Published:** 1948

**Authors:** 


### THIOURACIL IN THYROTOXICOSIS

A summary of the main points of Professor Himsworth's opening address in the Section of Medicine at the Annual Meeting of the British Medical Association at Cambridge, June 30th, 1948. With acknowledgments to the *British Medical Journal*, 1948, ii, p. 61.

---

It is now established that thiouracil can control the manifestations of thyrotoxicosis : patients vary widely in the speed of their response, but the majority are controlled within six weeks. No case is too severe for thiouracil treatment : to this may be attributed the virtual disappearance of the highly fatal thyroid crisis. A few cases react very slowly; those with nodular goitres and those previously treated with iodine. Omit iodine for a month before giving thiouracil : once thiouracil control is established iodine may be given.

*Dosage.*—Unnecessarily large doses have been used : a good response is obtained from 200 mgm. methyl thiouracil daily. Once symptoms are controlled, 25, 50—at most 100 mgm.—is sufficient ; more may produce myxoedema.

*Exophthalmos* (sclera showing below cornea), is found in only one-third of those with thyrotoxicosis and diffuse goitre, and in less than one-tenth of those with nodular goitre : in the majority the *appearance* of exophthalmos is due to lid retraction (sclera showing only above cornea). Thiouracil does not reduce true exophthalmos, but usually improves lid retraction.

*Goitre.*—The goitre may enlarge during treatment (over-dosage): some diminution in size is usual, but disappearance is rare. Nodular goitres remain unchanged.

*Fibrillation.*—Intermittent arrhythmia usually disappears : persistent arrhythmia is abolished by quinide after the thyrotoxicosis is controlled by thiouracil.

*Cure.*—Treatment must be continued for months after control is established: in patients who have had two years' remission after cessation of treatment, this had lasted 9–21 months ; of patients treated thirty months, two-thirds remain free from symptoms after cessation, and all in this group are well and at work.

*Dangers.*—In many patients the leucocyte-count drops during the first week of treatment : this can be ignored provided the total is not below 3000 with 50 per cent. polymorphs. About 10 per cent. of patients show idiosyncrasy, between sixth day and sixth week—

drug fever, rashes : continue treatment unless the polymorph count falls below danger point. Adenitis, lymphatic, salivary, etc., may be ignored. Agranulocytosis was seen in two per cent. of all cases treated: of these, one-quarter died. The lower doses now used and penicillin should reduce mortality : the polymorphs reappear within ten days of stopping thiouracil. Patients must be warned to stop the drug at once if they get fever or sore throat, and to call the doctor.

*Surgery or Thiouracil.*—Even the early figures given above show a mortality considerably less than that of the best surgeons : and included cases too bad for surgery. Thiouracil will do anything that surgery can : it is too early to judge permanence of results, but about one-third of the patients relapse sooner or later after the drug is stopped—another course may be given or the patient may prefer surgery.

